# Exposure to Radiofrequency Electromagnetic Fields Enhances Melanin Synthesis by Activating the P53 Signaling Pathway in Mel-Ab Melanocytes

**DOI:** 10.3390/ijms252212457

**Published:** 2024-11-20

**Authors:** Ju Hwan Kim, Dong-Jun Kang, Jun Young Seok, Mi-Hye Kim, Dong-Seok Kim, Sang-Bong Jeon, Hyung-Do Choi, Jung Ick Moon, Nam Kim, Hak Rim Kim

**Affiliations:** 1 Department of Pharmacology, College of Medicine, Dankook University, Cheonan 31116, Republic of Korea; jhkim731@dankook.ac.kr (J.H.K.); kdu1313@naver.com (D.-J.K.); seogant95@naver.com (J.Y.S.); 2 Department of Medical Laser, Graduate School, Dankook University, Cheonan 31116, Republic of Korea; mhhljizmfh@naver.com; 3 Department of Biochemistry, College of Medicine, Chung-Ang University, Seoul 06974, Republic of Korea; ds_kim@cau.ac.kr; 4 Radio and Broadcasting Technology Laboratory, ETRI, Daejeon 34129, Republic of Korea; sbjeon@etri.re.kr (S.-B.J.); choihd@etri.re.kr (H.-D.C.); jungick@etri.re.kr (J.I.M.); 5 School of Electrical and Computer Engineering, Chungbuk National University, Cheongju 28644, Republic of Korea; namkim@chungbuk.ac.kr

**Keywords:** radiofrequency electromagnetic fields, melanogenesis, phospho-CREB, MITF, tyrosinase, p53, MC1R

## Abstract

The skin is the largest body organ that can be physiologically affected by exposure to radiofrequency electromagnetic fields (RF-EMFs). We investigated the effect of RF-EMFs on melanogenesis; Mel-Ab melanocytes were exposed to 1760 MHz radiation with a specific absorption rate of 4.0 W/kg for 4 h/day over 4 days. Exposure to the RF-EMF led to skin pigmentation, with a significant increase in melanin production in Mel-Ab melanocytes. The phosphorylation level of cAMP response element binding protein (CREB) and the expression of microphthalmia-associated transcription factor (MITF), which regulate the expression of tyrosinase, were significantly increased in Mel-Ab after RF-EMF exposure. Interestingly, the expression of tyrosinase was significantly increased, but tyrosinase activity was unchanged in the RF-EMF-exposed Mel-Ab cells. Additionally, the expression of p53 and melanocortin 1 receptor (MC1R), which regulate MITF expression, was significantly increased. These results suggest that the RF-EMF induces melanogenesis by increasing phospho-CREB and MITF activity. Importantly, when Mel-Ab cells were incubated at 38 °C, the melanin production and the levels of tyrosinase significantly decreased, indicating that the increase in melanin synthesis by RF-EMF exposure is not due to a thermal effect. In conclusion, RF-EMF exposure induces melanogenesis in Mel-Ab cells through the increased expression of tyrosinase via the activation of MITF or the phosphorylation of CREB, which are initiated by the activation of p53 and MC1R.

## 1. Introduction

Owing to the rapid development of modern information and communication technology, humans are frequently exposed to electromagnetic fields. Therefore, there has been increasing public concern regarding the potential biological effects of radiofrequency electromagnetic fields (RF-EMFs) generated by mobile phones on the human body.

Because the skin is the outermost organ, it functions as an anatomical barrier against various external harmful environmental factors [1]. UV exposure is well known to cause hyperpigmentation and skin aging, including the formation of skin wrinkles [2,3]. The skin is often exposed to RF-EMFs, and may be physiologically affected by RF-EMF insults.

Skin pigmentation is induced by melanin synthesis (melanogenesis) in pigment-producing cells called melanocytes [4]. Melanin production is initiated and regulated by various signaling systems and transcription factors, including microphthalmia-associated transcription factor (MITF) and cyclic adenosine monophosphate (cAMP) response element-binding protein (CREB) [5], which regulates the transcription of the major pigmentation enzyme known as tyrosinase [5]. Tyrosinase is the initial enzyme in the melanin synthesis pathway and catalyzes the conversion of L-tyrosine to L-dihydroxyphenylalanine (L-DOPA) [5]. Melanocortin 1 receptor (MC1R), which is involved in regulating mammalian skin, is located on the plasma membrane of melanocytes, and binds to α-melanocyte-stimulating hormone (α-MSH) to regulate the expression of MITF, which ultimately upregulates melanin production [6]. Furthermore, p53 serves as a key regulator of melanogenesis by regulating MITF and tyrosinase expression in human melanocytes [7].

Recently, Kim et al. showed that exposure to RF-EMFs (both 1.76 GHz and 28 GHz) did not affect melanin synthesis or skin pigmentation in murine and human melanoma cells [8]. However, they found that the expression of genes related to melanin synthesis was increased by RF-EMF exposure. An extremely low-frequency electromagnetic field (ELF-EMF) increases the activity of ERK and p38, resulting in melanin synthesis, thereby inducing skin pigmentation in human melanocytes [9]. Exposure to 900 MHz RF-EMF led to changes in the expression of several proteins in human skin [10].

Skin is very important in protecting our body from various external stimulation. Among these protective mechanisms is the regulation of melanin production. The mechanism of the regulation of melanin production by exposure to electromagnetic fields has not yet been clearly explained. In this study, we aim to investigate the potential induction of skin pigmentation by RF-EMF exposure, a protective mechanism employed by melanocytes, using Mel-Ab cells.

## 2. Results

### 2.1. RF-EMF Exposure Significantly Increased the Number of Darkened Mel-Ab Cells and the Melanin Content

**Figure 1:**
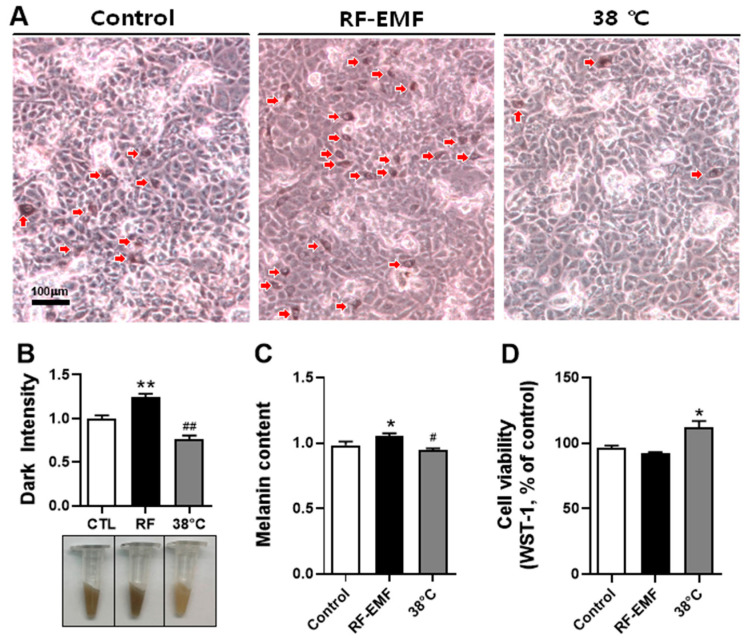
Cellular morphology and pigmentation of Mel-Ab melanocytes after RF-EMF exposure. The Mel-Ab cells were cultured for 4 d with or without 1760 MHz radiofrequency electromagnetic field (RF-EMF) exposure (at 4.0 W/kg for 4 h/d) and heat treatment (38 °C). (**A**) Cellular morphology was observed using a microscope (Olympus, CKX53, Tokyo, Japan). Red arrows indicate darkened Mel-Ab cells. The 200× magnified photos with 100 µm scale bar are shown. (**B**) The cell pellets of control, RF-EMF-exposed, and 38 °C-incubated Mel-Ab melanocytes (lower panel) and dark intensity of each pellet was quantified using the ImageJ software bundled with 64-bit Java 8 (upper panel). (**C**) Melanin content was measured in Mel-Ab cells under each condition. (**D**) Cell viability of Mel-Ab cells under each condition. Data are expressed as mean ± standard error of the mean. * *p* and # *p* < 0.05, ** *p* and ## *p* < 0.01 compared to control (*n* = 6). RF-EMF, radiofrequency electromagnetic field.

To elucidate the effect of RF-EMF on melanin synthesis in Mel-Ab melanocytes, the cells were exposed to a 1760 MHz RF-EMF at SAR 4.0 W/kg for 4 h/d over 4 d. Further, to determine whether the exposure to RF-EMFs includes thermal effects, the Mel-Ab melanocytes were heat-treated at 38 °C in an incubator. Cell images captured using a microscope revealed that RF-EMF exposure increased the number of darkened cells compared to the control group, but fewer dark cells were observed in the heat-treated sample (Figure 1A). The RF-EMF-exposed cells were darker, but the heat-treated cells were lighter than the sham-control cells (Figure 1B, lower panel), indicating that the intensity of dark coloration significantly increased in RF-EMF-exposed cells but decreased in the heat-treated cells (Figure 1B, upper panel). Next, the amount of intracellular melanin in the Mel-Ab melanocytes was quantified. The results showed that the melanin content significantly increased in the RF-EMF-exposed cells, but decreased in the heat-treated cells (Figure 1C). These results indicate that exposure to RF-EMFs can upregulate melanogenesis, but heat treatment at 38 °C downregulates melanogenesis in Mel-Ab cells. Finally, the proliferation of Mel-Ab cells was evaluated using the WST-1 assay after RF-EMF exposure. The viability of the Mel-Ab cells following RF-EMF exposure did not significantly change compared to the controls (Figure 1D).

### 2.2. Heat Shock Proteins Were Not Affected by RF-EMF Exposure in Mel-Ab Cells

**Figure 2:**
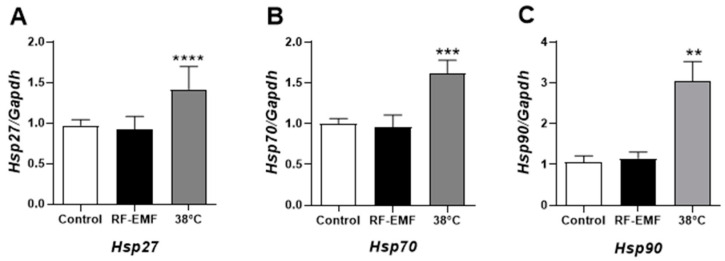
Expression levels of heat shock proteins in Mel-Ab melanocytes after RF-EMF exposure. Expression of *Hsp27* (**A**), *Hsp70* (**B**), and *Hsp90* (**C**) mRNA transcripts determined by quantitative real-time PCR. The relative mRNA levels of heat shock proteins were calculated by normalizing to the expression of *Gapdh*, using the 2^−ΔΔCt^ method. Data are expressed as mean ± standard error of the mean. ** *p* < 0.01, *** *p* < 0.001, **** *p* < 0.0001 compared to control (*n* = 4). RF-EMF, radiofrequency electromagnetic field.

The expression levels of heat shock proteins (HSPs) were examined to determine whether RF-EMF exposure induces thermal effects in Mel-Ab melanocytes. There were no significant differences in the mRNA levels of *Hsp27* (A), *Hsp70* (B), or *Hsp90* (C) in Mel-Ab cells (Figure 2). In addition, to confirm thermal effects on Mel-Ab melanocytes, cells were treated with 38 °C heat for 4 h/d over 4 d. After this treatment, the expression levels of *Hsp27* (A), *Hsp70* (B), and *Hsp90* (C) transcripts significantly increased in the Mel-Ab cells (Figure 2). These results suggest that the Mel-Ab cells exposed to RF-EMF were not affected by the possible thermal effects of RF-EMF exposure. 

### 2.3. RF-EMF Exposure Significantly Increased MITF Expression and CREB Phosphorylation in Mel-Ab Cells

**Figure 3:**
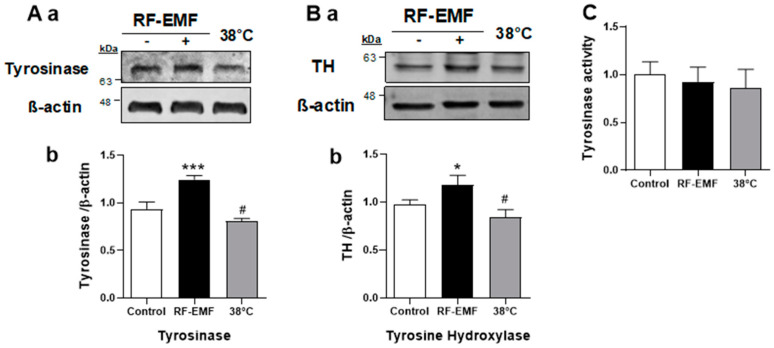
Expression levels of tyrosinase and tyrosine hydroxylase were increased by RF-EMF exposure. (**a**). Expressional quantification of tyrosinase (**A**) and tyrosine hydroxylase (**B**) using Western blotting. (**b**). The graphs show the quantification of protein levels of tyrosinase (**A**), and tyrosine hydroxylase (**B**) normalized to that of β-actin. (**C**). Tyrosinase activity was measured in each condition. The data indicate the mean ± standard error of the mean. Levels of statistical significance were evaluated using one-way ANOVA. *** *p* < 0.001, * *p* and # *p* < 0.05 compared to the control (*n* = 4). RF-EMF, radiofrequency electromagnetic field; TH, tyrosine hydroxylase.

MITF regulates the transcription of the major pigmentation enzyme known as tyrosinase [8]. To elucidate the effect of RF-EMF on melanogenesis in Mel-Ab cells, the cells were exposed to RF-EMF and heat-treated at 38 °C for 4 h/d over 4 d, and the expression levels of tyrosinase and TH were analyzed by Western blotting. The results showed that the levels of tyrosinase and TH enzymes were significantly increased in Mel-Ab cells after RF-EMF exposure, but were slightly decreased by heat treatment (Figure 3A,B). In addition, the intracellular tyrosinase activity was tested under each condition. However, the tyrosinase activity did not change under either condition (Figure 3C). These results suggest that the regulation of tyrosinase and TH expression could explain the increased melanogenesis in Mel-Ab cells after RF-EMF exposure.

### 2.4. Expression of p53 and Mc1r Significantly Increased by RF-EMF Exposure

**Figure 4:**
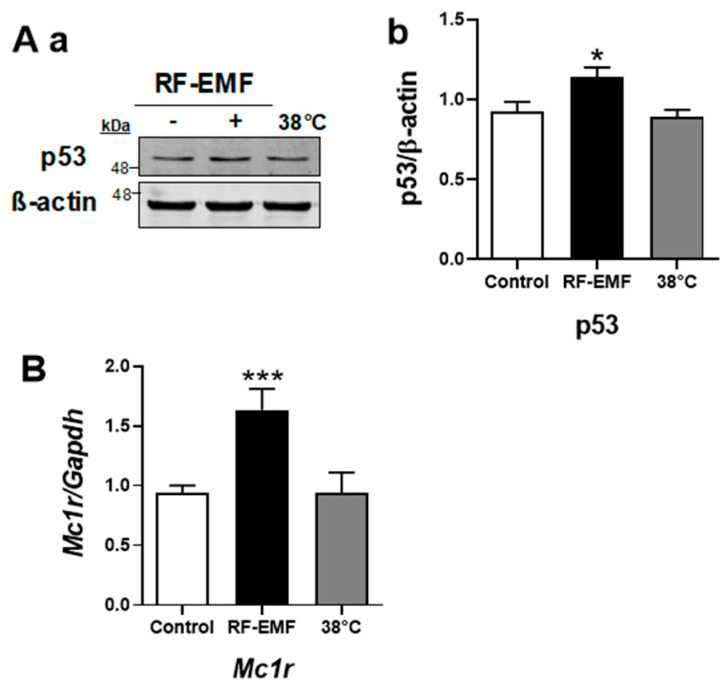
Expression levels of p53 and *Mc1r* were significantly increased by RF-EMF exposure in Mel-Ab melanocytes. (**A**). Expressional quantification of p53 by Western blotting (**a**). The graphs show the quantification of protein levels of p53 normalized to those of β-actin (**b**). (**B**). The mRNA levels of *Mc1r* were analyzed by quantitative real-time PCR. The relative mRNA levels of *Mc1r* were calculated by normalizing to the expression of *Gapdh* by the 2^-ddCt^ method. Data are expressed as the mean ± standard error of the mean. * *p* < 0.05, *** *p* < 0.001 compared to control (*n* = 3). RF-EMF, radiofrequency electromagnetic field.

**Figure 5:**
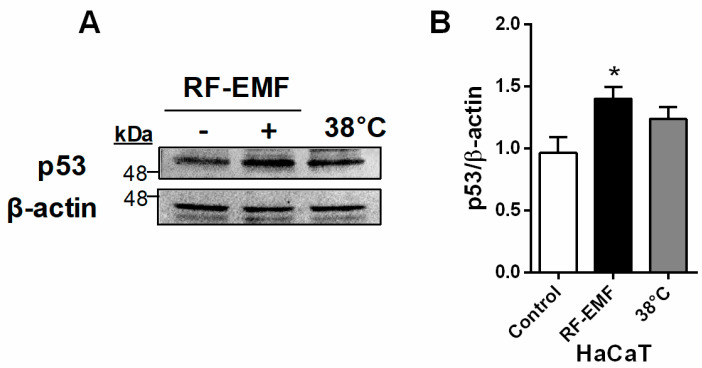
The expression level of p53 was significantly increased by RF-EMF exposure in HaCaT keratinocytes. HaCaT cells were exposed to RF-EMF or treated with 38 °C heat. (**A**). Expressional quantification of p53 using Western blotting. (**B**). The graphs show the quantification of protein levels of p53 normalized to those of β-actin. Data are expressed as the mean ± standard error of the mean. * *p* < 0.05 compared to control (*n* = 3). RF-EMF, radiofrequency electromagnetic field.

The expression of p53 was significantly increased by RF-EMF exposure in both Mel-Ab (Figure 4A) and HaCaT cells (Figure 5). In addition, the expression level of *Mc1r* mRNA was significantly increased by RF-EMF exposure in Mel-Ab cells (Figure 4B). However, there were no changes in the heat-treated Mel-Ab or HaCaT cells. These data suggest that the increased expression levels of p53 and *Mc1r* following RF-EMF exposure initiate increased melanogenesis in Mel-Ab cells.

## 3. Discussion

The skin is the largest organ and outermost layer of the body [1], and it is frequently exposed to various environmental stresses, such as UV radiation, chemicals, extreme temperatures, and electromagnetic fields. Upon exposure to various external stimuli, the skin exhibits various responses, such as the formation of wrinkles, loss of moisture, and hypo- or hyperpigmentation. Generally, melanin plays an important role in preventing skin damage caused by UV radiation, which leads to hyperpigmentation due to the excess production of melanin. Hyperpigmentation is a well-known response to excess UV exposure [11] and therapeutic drugs, such as nonsteroidal anti-inflammatory or antihypertensive agents [12].

In this study, we studied the possible effect of 4G LTE 1760 MHz RF-EMF at SAR 4.0 W/kg on Mel-Ab cell pigmentation when irradiated for 4 h/d over 4 d. Mel-Ab cells are a spontaneously immortalized melanocyte cell line derived from the skin melanocytes of C57BL/6 mice. These cells produce significant amounts of melanin during culture, making them a valuable model for studying skin pigmentation and evaluating anti-melanin agents. Presently, we found that RF-EMF exposure could cause skin pigmentation by significantly increasing melanin production in Mel-Ab cells. Through this, we may indirectly infer potential effects on human skin.

After exposure to RF-EMF, the Mel-Ab cells showed significantly increased melanin content (Figure 1A,B). Despite the significant difference in frequency, these data are consistent with previous report that 60–75 Hz ELF-EMF exposure led to upregulated melanin synthesis visualized by silver staining in B16F10 melanoma cells, and the cells exposed to ELF-EMF in tubes were darker than the control cells [13].

Our results indicated that upregulated melanogenesis led to an increase in melanin content in Mel-Ab melanocytes after exposure to the RF-EMF (Figure 1C). This is consistent with the finding that the increased melanin content observed in melanocytes indicates increased darkening of cells due to more melanin, as reported by Hu [14]. However, 1N NaOH was added to solubilize melanin from the cells in this study, but it is important to note that under these conditions, there exists a potential risk of melanin degradation [15]. Therefore, these data suggest that exposure to a 1760 MHz RF-EMF may activate the melanogenic pathway in Mel-Ab cells. Therefore, we investigated the possible pathways that induce melanin synthesis in response to RF-EMFs. Darkened skin color due to increased epidermal melanin by ultraviolet (UV) irradiation is a well-studied response of human skin [16]. 

**Figure 6:**
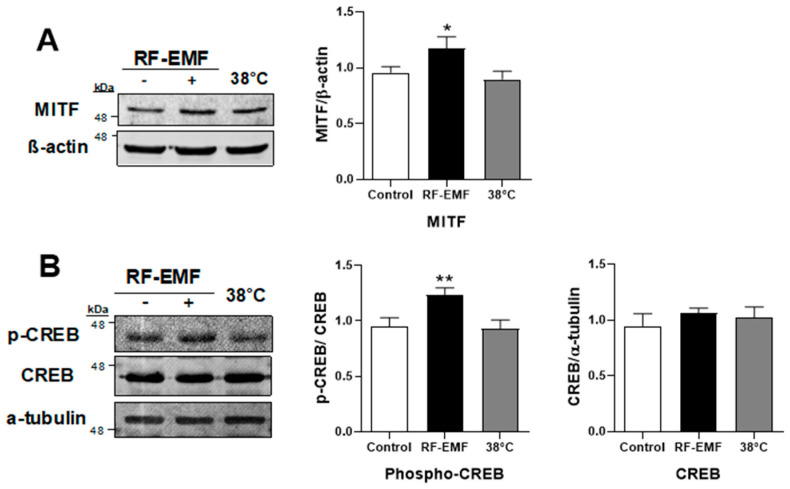
Expression levels of MITF and phospho-CREB were increased by RF-EMF exposure. Quantification of MITF (**A**), p-CREB (Ser133), and CREB (**B**) expression by Western blotting. Data are presented as mean ± standard error of the mean. Statistical significance was evaluated using one-way ANOVA; * *p* < 0.05, ** *p* < 0.01 compared to control (*n* = 6). RF-EMF, radiofrequency electromagnetic field.

Briefly, excessive UV irradiation activates p53, which leads to the transcriptional upregulation of proopiomelanocortin (POMC) [17]. POMC activates α-melanocyte-stimulating hormone (α-MSH) [17]. α-MSH binds to melanocortin 1 receptors (MC1R), which causes cAMP production, and cAMP leads to the phosphorylation of CREB, an important MITF promoter, which, in turn, promotes the activation of MITF transcription factor [18]. In particular, CREB requires phosphorylation at Ser133 to activate transcription [19]. Our results showed that the expression level of CREB was not changed, but was highly phosphorylated at Ser133 (Figure 6B), indicating that MITF was also significantly increased in Mel-Ab melanocytes after RF-EMF exposure (Figure 6A). Enhanced MITF then binds to the E-box sequences in the promoter regions of melanin production genes and upregulates the transcription of tyrosinase, which is the rate-limiting enzyme for melanogenesis [17,18,20].

Interestingly, the expression level of tyrosinase was augmented in melanocytes following RF-EMF exposure (Figure 3A), whereas tyrosinase activity did not change in Mel-Ab cells (Figure 3C). In addition, TH, which catalyzes the hydroxylation of tyrosine to L-DOPA [21], was increased by RF-EMF exposure (Figure 3B). Tyrosine hydroxylase isoform I is present in the melanosome membrane adjacent to tyrosinase and promotes the activation of tyrosinase by catalyzing the conversion of L-tyrosine to L-DOPA [21]. These enzymes upregulated and activated melanin synthesis in Mel-Ab melanocyte following 1760 MHz RF-EMF exposure.

Conversely, Mel-Ab cell pigmentation was reduced by heat treatment at 38 °C in these cells compared to the control melanocytes (Figure 1). RF-EMF exposure is well known to exert thermal effects on the body, based on the theory that when electromagnetic waves are absorbed by a living body, the energy of electrons vibrates ions or dipole molecules, potentially generating heat in tissues [22,23].

Previously, other research groups demonstrated that heat treatment in Mel-Ab melanocytes reduced melanogenesis in a temperature-dependent manner by showing that heat treatment decreases melanin content and eventually inhibits MITF promoter activity and the tyrosinase level in Mel-Ab cells [24]. Our results showed that heat treatment at 38 °C in Mel-Ab melanocytes downregulated melanogenesis, as evidenced by decreases in the number of darkened cells, the intensity of cell pellet coloration, and the amount of intracellular melanin in the heat-treated Mel-Ab cells (Figure 1). In addition, the expression level of tyrosinase was decreased in the 38 °C heat-treated Mel-Ab cells (Figure 3A).

Furthermore, we studied the expressional changes in *HSP27, HSP70*, and *HSP90* in Mel-Ab cells (Figure 2). However, *HSPs* expression was not affected by RF-EMF exposure in the present study. Meanwhile, the cells treated at 38 °C showed significant increases in *HSP27, HSP70,* and *HSP90*.

In addition, p53, an upstream activator of melanogenesis, was increased in the Mel-Ab melanocytes (Figure 4A), thereby increasing *Mc1r* transcripts in melanocytes after exposure to RF-EMF (Figure 4B). As mentioned above, UV exposure indicates that α-MSH production is regulated in keratinocytes by p53 via a p53 consensus sequence in the POMC gene promoter [25]. POMC activates α-MSH, which binds to MC1R, leading to the phosphorylation of CREB [17]. However, in the absence of keratinocytes, there is a strong p53-mediated melanogenic response to UV radiation in melanocytes in vitro, indicating that p53 regulates the transcription of hepatocyte nuclear factor 1α (HNF1α), which is a tyrosinase transcription factor in melanocytes [26]. In addition, it is difficult to co-culture keratinocytes and melanocytes because of the differing culture conditions needed. We tested the expression level of p53 in keratinocytes using HaCaT cells in separate cultures following RF-EMF exposure and heat treatment at 38 °C (Figure 5). We found that the p53 transcription factor expression also increased in HaCaT keratinocytes after RF-EMF exposure. Thus, these data strongly suggest that melanin synthesis in Mel-Ab melanocytes after exposure to RF-EMF is initiated by the upregulation of p53 in keratinocytes. As a key regulator of melanogenesis, p53 upregulation activates melanin synthesis by controlling MITF and tyrosinase expression, which then leads to hyperpigmentation in Mel-Ab melanocytes after RF-EMF exposure.

## 4. Materials and Methods

### 4.1. Cell Culture

The Mel-Ab cell line (gifted from Amore Pacific Corp. (Seoul, Korea) to D.S.K.) is a mouse-derived spontaneously immortalized melanocyte cell line that synthesizes large amounts of melanin [27]. The Mel-Ab cells were maintained in Dulbecco’s modified Eagle’s medium (DMEM) supplemented with 10% fetal bovine serum (FBS), 100 nM 12-O-tetradecanoylphorbol-13-acetate (TPA) (Sigma-Aldrich, St. Louis, MO), 1 nM cholera toxin (CT) (Sigma-Aldrich, St. Louis, MO), 50 µg/mL streptomycin, and 50 µg/mL penicillin at 37 °C in 5% CO_2_. Cell numbers were automatically measured both before RF-EMF exposure on the first day and the next day after RF-EMF exposure on the 4th day using a specific program (CKX-CCSW version 1. 1. 1. 29 software, Olympus, Tokyo, Japan).

### 4.2. RF-EMF Cell Exposure System

The in vitro radiofrequency radiation exposure device uses a radial transmission line exposure system that can simultaneously expose multiple cells [28]. In this study, a 1760 MHz RF-EMF LTE signal was applied to the radial transmission line exposure system after amplification. The maximum input power was 60 W, and the exposure level and time were adjusted by manipulating the controls. The exposure signal is fed through a conical antenna with broadband characteristics. The external dimensions of the RF-EMF generator are 843 mm × 825 mm × 315 mm. The chamber is made of aluminum, which serves as an electromagnetic shield. The exposure system was specifically designed to control the environmental conditions, including ventilation, humidity, and temperature. The gas from the incubator was circulated throughout the chamber to maintain CO_2_ density and humidity inside the chamber. To maintain the medium temperature at 37 °C in culture dishes or flasks during RF-EMF exposure, a water pump was used to circulate water throughout the bottom of the cavity. Information on this device has been previously described in detail [29]. After RF-EMF irradiation, the cells were immediately transferred and incubated in a cell culture incubator. RF-EMF-exposed cells and unexposed cells were collected 1 d after the last exposure.

### 4.3. Cell Proliferation Assay

Cell proliferation was estimated using a colorimetric method based on water-soluble tetrazolium salts (WST-1) (CellVia, AbFrontier, Seoul, Korea) according to the manufacturer’s instructions. Briefly, 5 × 10^4^ cells/well were placed in a 96-well microplate and incubated overnight. Then, the cells were exposed to a 1760 MHz RF-EMF according to the following schedule: SAR 4.0 W/kg, 4 h/d over 4 d. Then, 24 h after RF-EMF exposure on the 4th day, 10 μL of WST-1 reagent was added to 100 μL of culture medium in each well, and incubation was continued for 2 h. The absorbance of the samples was measured at 440 nm using a 96-well microplate spectrophotometer (Multiskan GO; Thermo Fisher Scientific, Waltham, MA). The assay was performed by transferring the assay reaction solution to a clean 96-well plate to exclude the possibility that the melanin pigment of Mel-Ab was participating in the absorbance observed at 440 nm.

### 4.4. Measurement of Melanin Content

The melanin content of the cultured Mel-Ab cells was measured as previously described [30]. After RF-EMF exposure or heat treatment at 38 °C, the cells were collected by trypsinization and subsequent centrifugation, and then washed with phosphate-buffered saline (PBS). The cell pellets were dissolved in 0.5 mL of 1 M NaOH containing 10% (*v/v*) of DMSO, incubated for 30 min at 100 °C in a heat block, and then centrifuged at 16,000 g for 20 min. The optical density of the supernatant was measured at 400 nm using a microplate reader. The melanin content was calculated using the following equation, calculated as the percentage of the difference in absorbance between the RF-EMF exposure and control groups:

Melanin content (%) = (absorbance of RF-EMF exposed group)/(absorbance of control group)

### 4.5. Tyrosinase Activity

Tyrosinase activity was analyzed as previously described [30]. Mel-Ab cells were cultured in 6-well dishes. After 4 d RF-EMF exposure or heat treatment at 38 °C, the cells were washed with ice-cold PBS and lysed with 0.1 M phosphate buffer (pH 6.8) containing 1% Triton X-100 (Sigma-Aldrich, St. Louis, MO, USA). The lysed cells were collected by scraping. The cells were then disrupted by freezing and thawing and the lysates were clarified by centrifugation at 20,000× *g* for 10 min at 4 °C. After quantifying the protein levels and adjusting the concentration with lysis buffer, 90 μL of each lysate containing the same amount of protein was placed in each well of a 96-well plate, and 10 μL of 10 mM L-DOPA was added to each well. The control wells contained 90 μL lysis buffer and 10 μL 10 mM L-DOPA. After incubation at 37 °C, absorbance was measured every 10 min for at least 1 h at 475 nm using a microplate reader.

### 4.6. Immunoblotting

Mel-Ab cells were lysed using RIPA buffer (Thermo Fisher Scientific, Waltham, MA, USA) supplemented with protease and phosphate inhibitor cocktail (Thermo Scientific, Waltham, MA, USA). Whole-cell lysates were briefly sonicated. Protein concentrations were measured using a Bio-Rad DC™ protein assay (Bio-Rad, Hercules, CA, USA) and total protein (20–50 μg) was separated by 10% sodium dodecyl sulfate–polyacrylamide gel electrophoresis (SDS-PAGE) and transferred to a polyvinylidene difluoride (PVDF) transfer membrane (Bio-Rad, Hercules, CA, USA).

MITF, phospho-CREB, CREB, tyrosinase, tyrosine hydroxylase (TH), p53, α-tubulin, and β-actin were detected in the membranes using antibodies to MITF (1:1000, #ab20663; Abcam, Cambridge, UK), CREB (1:1000, #9104; Cell Signaling Technology, Danvers, MA, USA), phospho-CREB (Ser133) (1:1000, #8763; Cell Signaling Technology, Danvers, MA, USA), tyrosinase (1:500, #sc-20035; Santa Cruz, Dallas, TX, USA), tyrosine hydroxylase (1:500, #T2928; Sigma-Aldrich, St. Louis, MO), p53 (1:500, #9282; Cell Signaling Technology, Danvers, MA, USA), α-tubulin (1:3000, #sc-23948; Santa Cruz, Dallas, TX, USA), and β-actin (1:3000, #A5441; Sigma-Aldrich, St. Louis, MO, USA). Protein bands were visualized using an Odyssey infrared imaging system (Li-Cor Biosciences, Lincoln, NE, USA). The intensity of each band was quantified and normalized using α-tubulin or β-actin as internal loading controls. 

### 4.7. Quantitative Real-Time RT-PCR

Quantitative real-time PCR (qRT-PCR) was performed following a published method [31]. The total cellular RNA was extracted from the Mel-Ab cells using Ambion TRIzol reagent (Life Technologies, Foster City, CA, USA). The RNA concentrations were determined by measuring absorption at 260 nm using a μDropTM (Thermo Fisher Scientific, Waltham, MA, USA). After RNA quantification, 2 μg of total RNA was used for reverse transcription PCR using AccuPower CycleScript RT PreMix dT20 (Bioneer, Daejeon, Republic of Korea), according to the manufacturer’s protocol. For qRT-PCR, the synthesized cDNA and primers were prepared using the SYBR Green Master Mix (QIAGEN, Hilden, Germany). The primer sequences were as follows: *Mc1r*: forward 5′-TGGGC ATCAT TGCTA TAGAC-3′ and reverse 5′-AACGG CTGTG TGCTT GTAGT-3′; *Hsp27*: forward 5′-CCCAG TGAAT CCCCT GTCTA-3′ and reverse 5′-CCCCC AGGTT TTGGT TTATT-3′; *Hsp70*: forward 5′-TGCTG ATCCA GGTGT ACGAG-3′ and reverse 5′-CGTTG GTGAT GGTGT CTTG-3′; *Hsp90*: forward 5′-GGCAT CGATG AAGAT GAGGT-3′and reverse 5′-ACATG AGCAG AGAGC CAGGT-3′; and mouse *Gapdh* (QIAGEN, Hilden, Germany). qRT-PCR was performed and analyzed using Rotor-Gene Q with Rotor-Gene Q software v. 2.3.1 (QIAGEN, Hilden, Germany). Gene expression was normalized to that of *Gapdh* and analyzed using the 2^−ΔΔCt^ method. Furthermore, the expression values of the RF-EMF-exposed cells were normalized to those of the control cells.

### 4.8. Statistical Analysis

All data are presented as the mean ± SEM. The n values represent the number of independent samples used in the experiments. Statistical differences between groups were tested using one-way analysis of variance (ANOVA), and the cut-off for statistical significance was set at *p* < 0.05. GraphPad Prism 4 software (GraphPad Software, La Jolla, CA, USA) was used for the statistical analysis.

## 5. Conclusion

In summary, we have shown that 1760 MHz RF-EMF exposure (SAR of 4.0 W/kg for 4 h/d over 4 d) could induce hyperpigmentation by activating the melanin synthesis pathway in Mel-Ab melanocytes, suggesting that skin pigmentation could be affected by RF-EMF exposure in a way that is distinct from simple thermal effects.

## Data Availability

All data generated or analyzed during this study are included in the published article.
